# Resistance Profiles of Staphylococcus aureus and Immunological Status in Pregnant Women at Bafang, West Region of Cameroon: A Cross-Sectional Study

**DOI:** 10.7759/cureus.8648

**Published:** 2020-06-16

**Authors:** Ornella Joseline Tchokouaha Ngalani, Wiliane Jean Takougoum Marbou, Armelle Tsafack Mbaveng, Victor Kuete

**Affiliations:** 1 Biochemistry, Faculty of Science, University of Dschang, Dschang, CMR; 2 Biochemistry, Faculty of Sciences, University of Dschang, Dschang, CMR; 3 Biochemistry, University of Dschang, Dschang, CMR

**Keywords:** pregnancy, staphylococcus sp, immunological profile, mrsa

## Abstract

Background

*Staphylococcus aureus*, a facultative aero-anaerobic Gram-positive coccus typically considered normal ﬂora in the human gastrointestinal tract, have increasingly become a major cause of healthcare-associated infections over the past decade. This study aimed to evaluate the changes in immune factors in pregnant women colonized by *S. aureus* in the town of Bafang, West Region of Cameroon.

Methods

A cross-sectional study was carried in antenatal care unit in various health center in Bafang. *S. aureus* were isolated in stools using specific bacterial culture media. Antimicrobial susceptibility test was carried out using disk diffusion method. Blood was used to measure CD3, CD4 and CD8 T-cell lymphocyte counts, white blood cell count, high sensitivity C-reactive protein (hs-CRP) and interleukin-6 measurement using flow cytometry, optical detection and the ELISA solid phase direct sandwich method, respectively.

Results

Out of the 169 patients studied, 76.30% patients were pregnant women and 23.70% were non-pregnant women. *S. aureus* were isolated in 70.41% participants that is, 78.15% in pregnant and 21.85% in non-pregnant women. The mean age was significantly higher in non-pregnant women (29.38 ± 7.685 years) compared to pregnant women (25.55 ± 5.521 years). CD4 T-cell (574.80 ± 165.94; 754.03 ± 162.28, p < 0.001), were significantly lower in pregnant than non-pregnant women respectively, contrary for CD8 T-cell (333.86 ± 233.04; 250.40 ± 227.75, p = 0.043). *S. aureus* were significantly more isolated in pregnant women with a CD4 T-cell count between 410 and 625 cells/µl (p < 0.001). *S. aureus* were more susceptible to imipenem (91.40%), (100%); ciprofloxacin (65.59%), (69.44%); amikacin (96.77%), (100%) and resistant to chloramphenicol (78.49%), doxycycline (64.52%) and cefotaxime (51.61%) in pregnant women. *Staphylococcus aureus* showed a significant increased multidrug resistant (MDR) and methicillin-resistant *S. aureus *(MRSA) phenotypes in pregnant compared to non-pregnant women (p < 0.05).

Conclusion

The present study revealed that, *Staphylococcus aureus*, including resistant phenotypes should be considered in pregnant women to improve their health care.

## Introduction

*Staphylococcus aureus*, a facultative aero-anaerobic Gram-positive coccus typically considered normal ﬂora in the human gastrointestinal tract, have increasingly become a major cause of healthcare-associated infections over the past decade [1]. However, *S. aureus*, considered as the most common species of the genus Staphylococcus that causes staphylococcal infections in humans, has emerged as an important human pathogen [2]. The carriage of *S. aureus* is an important source of nosocomial infection and community acquired infections; and antibiotic-resistant infections due to this microorganism including but not limited to methicillin-resistant *S. aureus* (MRSA) have been previously reported to commonly colonize the throat, skin, and gastrointestinal tract of humans [3]. It has an impressive arsenal of virulence factors including toxins, proteases, nucleases but also various proteins allowing it to cling to tissues and escape the immune response [4]. Intestinal carriage of *S. aureus* has not been widely investigated despite its potential clinical impact [5]. The population at high risk of *S. aureus* infection except children consists of the elderly, HIV-infected patients, transplant patients and pregnant women [6]. There are limited data on *S. aureus* and MRSA carriage rates among pregnant women. More information about the epidemiologic condition of *S. aureus* carriage and infection in this population is urgently needed. During pregnancy the immune system of mother is altered with an enhanced humoral immune response and suppressed cell-mediated immunity [7].

Although many studies have been performed on pregnancy disease, the evaluation of immune parameters for the pathogenesis of *S. aureus* resisting methicillin is still unknown. In Cameroon, there is a paucity of data on this public health issue. Therefore, this study aimed to evaluate the changes in immune factors, in pregnant patients, in order to determine the antibiotic susceptibility patterns of *S. aureus*'s strains isolated at the Bafang Hospital, Cameroon.

## Materials and methods

Study framework

It was a cross-sectional epidemiological study over a period of three years (from November 2016 to September 2019) on all pregnant women without distinction of gestational age on prenatal consultation in the various health center of Bafang locality (West Region of Cameroon) such as AD LUCEM Banka Bafang, district hospital, Dokovie Bafang Annex Center. Pregnant women with hepatitis, HIV were not included in this study.

Biological materials

Blood and stool samples were taken from 169 participants who were attending the hospital for a bacteriological examination and with given informed consent. Sociodemographic data (age, marital status, gestational age, activity carried out, total number of pregnancies) were collected using a designed questionnaire. Duplicates were systematically eliminated.

Samples collection

In this study, 169 samples (blood and stool) were collected under aseptic conditions prior to antibiotic therapy and processed within two hours of reception. The spatula container was put in a sealed plastic bag and the patients washed their hands well with soap and water. Any leftover stool was flushed down in toilet. The sample was returned to the lab as soon as possible and the microbiology analyses done immediately. A quantity of 10 ml of collected blood was directly introduced into three tubes; two containing the anticoagulant (heparin) and one dry tube without anticoagulant. Upon reception of the samples, the blood samples were directly sent to the haematology and serology blocks for analysis of white blood cell count, C-reactive protein, interleukin-6 and CD4, CD3, CD8 T-lymphocytes, respectively. While stool samples were used for microbiological analyses.

Analysis of blood samples

The blood in the heparin tube was analyzed after being homogenized to avoid the formation of blood clots using a cell counter (Mindray PE 6800®, PROCAN, China, (Mainland)) for the white blood cell count; the automated cell counter directly calculates and gives the results of total white blood cells, lymphocytes, monocytes, and granulocytes. The counting of CD4, CD3 and CD8 T-lymphocytes was carried out by the flow cytometry using the FACSCalibur (Becton Dickinson, San Jose, CA, USA). Depending on the number of cells obtained, the cells were stained at room temperature for 30 minutes with 100 ml of anti-CD4 and PE-anti-CD8 monoclonal antibodies labeled with fluorescein isothiocyanate, then washed once in the FACS buffer. The surface expression of CD4 and CD8 was defined by the flow cytometry. Lymphocytes were defined by the forward and sideward diffusion parameter, and they were controlled in such a way that cells in the defined window could be taken into consideration. The flow cytometry data were analysed within 24 hours of staining, using software (flow Joe). The results were expressed as percentage of positive flow cytometry for CD4 and CD8. The measurement of interleukin-6 (IL-6) using the enzyme-linked immunosorbent assay (ELISA) kits (R & D system, London, UK), was based on the manufacturer’s instructions. The blood collected in the dry tube was analysed by spectrophotometry for the quantification of the C-reactive protein (CRP) (Actim CRP®, the Medix Biochemica® company, Finland). The hs-CRP was measured in duplicate by a high sensitivity enzyme-linked immunosorbent assay (ELISA), as initially described above. The sensitivity of the assay was 0.2 ng/ml. Amounts of 3.9% and 7.4% were obtained for the intra- and the inter-trial variability, respectively.

Isolation and identification of *Staphylococcus aureus* from stool

The clinical specimens were inoculated onto plates of mannitol salt agar (MSA); they were incubated at 37°C for 24 h. All colonies from primary culture were purified by subculturing onto freshly prepared MSA medium and incubating at 37°C for 24 h to 48 h [8]. The smear was prepared from the isolated culture on clean grease-free microscopic glass slide and stained with Gram's method of staining. The stained smear was observed under the microscope. Smear revealed Gram positive, spherical cells arranged in irregular clusters resembling to bunch of grapes. Biochemical tests were performed to confirm *S. aureus* using coagulase positive, catalase positive, and they produce yellowish colonies on MSA [9].

Antibiotics susceptibility testing

The susceptibility of isolates to different anti-microbial agents was done by disk diffusion method using commercial disks and interpreted according to EUCAST in 2014 [10]. The results were recorded as susceptible(S), susceptible dose dependent (SDD) and resistant (R). Pure colonies of *S. aureus* cultures were inoculated in peptone water and incubated at 37°C to get turbidity equal to 0.5 on the McFarland scale (108 CFU/ml). A sterile cotton swab was dipped into the inoculation, and the excess was removed by pressing the swab to the sides of the tube. The entire Mueller Hinton agar surface was swabbed. The inoculation was allowed to dry for 15 min. Antibiotic discs were applied on the medium. The plates were incubated at 37°C and examined after 18-24 h. The antimicrobial agents tested were the following: cefoxitin (CEFO, 30 µg), Imipénem (IMI, 10 µg), erythromycin (ERY, 15 µg), amikacine (AMI, 30 µg), ciprofloxacin (CIP, 5 µg), vancomycin (VAN, 30 µg), doxycycline (DOX, 25 µg), chloramphenicol (CHL, 30 µg). The susceptibility to methicillin was tested using a 30-µg cefoxitin disk. An inhibition halo < 25 mm was considered methicillin resistant [11].

Ethical clearance

Ethical clearance was obtained from The CAMBIN Ethics Review and Consultancy Committee (ERCC) on 24th April 2019 with the Reference number: CBI/437/ERCC/CAMBIN and then we obtained the approval of the Director of the Bafang Health Area. Authorization to collect samples was obtained from the different hospital. Informed consent was obtained from all the study participants.

Processing and statistical analysis of data

All statistical analyses were performed using SPSS software, version 20 (IBM Corp., Armonk, NY) and Microsoft Excel 2010. Categorical variables were compared via a chi-square or Fisher’s exact test, and continuous variables were compared with the Student t-test or Mann-Whitney U test, as appropriate. A p-value of <0.05 was considered statistically signiﬁcant for all comparisons.

## Results

Out of the 169 patients studied, 129 (76.30%) patients were pregnant women and 40 (23.70%) were non-pregnant women who were prescribed coproculture examinations by physicians. On the other hand, these patients included women that were couples (married, cohabiting) and single (single, separated, divorced, widowed). Table 1 shows that 82 (77.36%) pregnant women are couples compared to 24 (22.64%) non-pregnant women. Similarly, 47 (74.6%) pregnant women were single compared to 16 (25.4%) non-pregnant women. There exists a significant difference in the distribution of the different age groups. A significant number of pregnant women were observed in the age groups ranging from 22-30 (80.52%), followed by 14-21 (83.63%), and 31-32 (65.52%), respectively. The mean age of the participants was significantly higher in non-pregnant women (29.38 ± 7.685 years) compared to pregnant women (25.55 ± 5.521 years).

**Table 1:** Demographic profile of patients according to the pregnant status. NA: Not Applicable

Parameters		Pregnant Women, n = 129 (%)	Non-pregnant Women, n = 40 (%)	p-value
Gestational age (trimester)	First	39 (30.23)	NA	NA
	Second	47 (36.43)		
	Third	43 (33.33)		
Matrimonial status	Couple (N = 106)	82 (77.36)	24 (22.64)	0.684
	Single (N = 63)	47 (74.6)	16 (25.4)	
Different age groups	14-21; (N = 55)	46 (83.63)	9 (16.36)	0.003
	22-30; (N = 77)	62 (80.52)	15 (19.48)	
	31-38; (N = 29)	19 (65.52)	10 (34.48)	
	39-47; (N = 8)	2 (25)	6 (75)	
Age average ± SD (min-max)	Total	25.55 ± 5.521 (14-40)	29.38 ± 7.685 (17-47)	0.001
	Couple	26.87 ± 5.175 (17-40)	32.21 ± 8.016 (19-47)	
	Single	23.26 ± 5.403 (14-37)	25.13 ± 4.787 (17-33)	

Table 2 shows the mean values of the blood tests obtained in the study population, as well as the white blood cell line, CD4, CD3 and CD8 T-lymphocytes, hs-CRP and interleukin-6. Results show that, CD4 T-cell (574.80 ± 165.94; 754.03 ± 162.28, p < 0.001) respectively, were significantly lower in pregnant than non-pregnant women, contrary to CD8 T-cell (333.86 ± 233.04; 250.40 ± 227.75, p = 0.043). Total white blood cells (p = 0.881), granulocytes (p = 0.720) were higher but non-significant in pregnant women compared to those who were non-pregnant.

**Table 2:** Mean values of the blood parameters obtained in the pregnant and non-pregnant women. SD: Standard Deviation; min: minimum; max: maximum

Parameters	Pregnant Women (n = 129)		Non-Pregnant Women (n = 40)		P-value
	Average values ± SD	Range [min-max]	Average values ± SD	Range [min-max]	
Total WBC (Cell x 10^3^/µl)	7.44 ± 3.05	1.80 - 23.10	7.51 ± 2.60	3.80 - 14.40	0.881
Lymphocytes (Cell x 10^3^/µl)	1.93 ± 0.96	0.10 - 6.30	2.16 ± 1.05	0.70 - 5.60	0.209
Monocytes (Cell x 10^3^/µl)	0.57 ± 0.48	0.10 - 4	0.65 ± 0.82	0.10 - 5.30	0.442
Granulocytes (Cell x 10^3^/µl)	4.90 ± 2.74	0.30 - 18.8	4.73 ± 2.31	1.30 - 11	0.720
CD4+ T cell (Cell/µl)	574.80 ± 165.94	193 - 1056	754.03 ± 162.28	390 - 1054	<0.001
CD3/CD4+ T cell (Cell/µl)	736.72 ± 348.13	91 - 1520	741.03 ± 335.97	91 - 1431	0.944
CD8+ T cell (Cell/µl)	333.86 ± 233.04	12 - 805	250.40 ± 227.75	12 - 722	0.043
hs-CRP (mg/l)	6.67 ± 6.81	0.10 - 50.20	6.92 ± 5.03	0.80 - 28.80	0.828
IL-6 (pg/ml)	110.48 ± 81.90	25 - 640	117.37 ± 107.70	25 - 480	0.668

**Figure 1:**
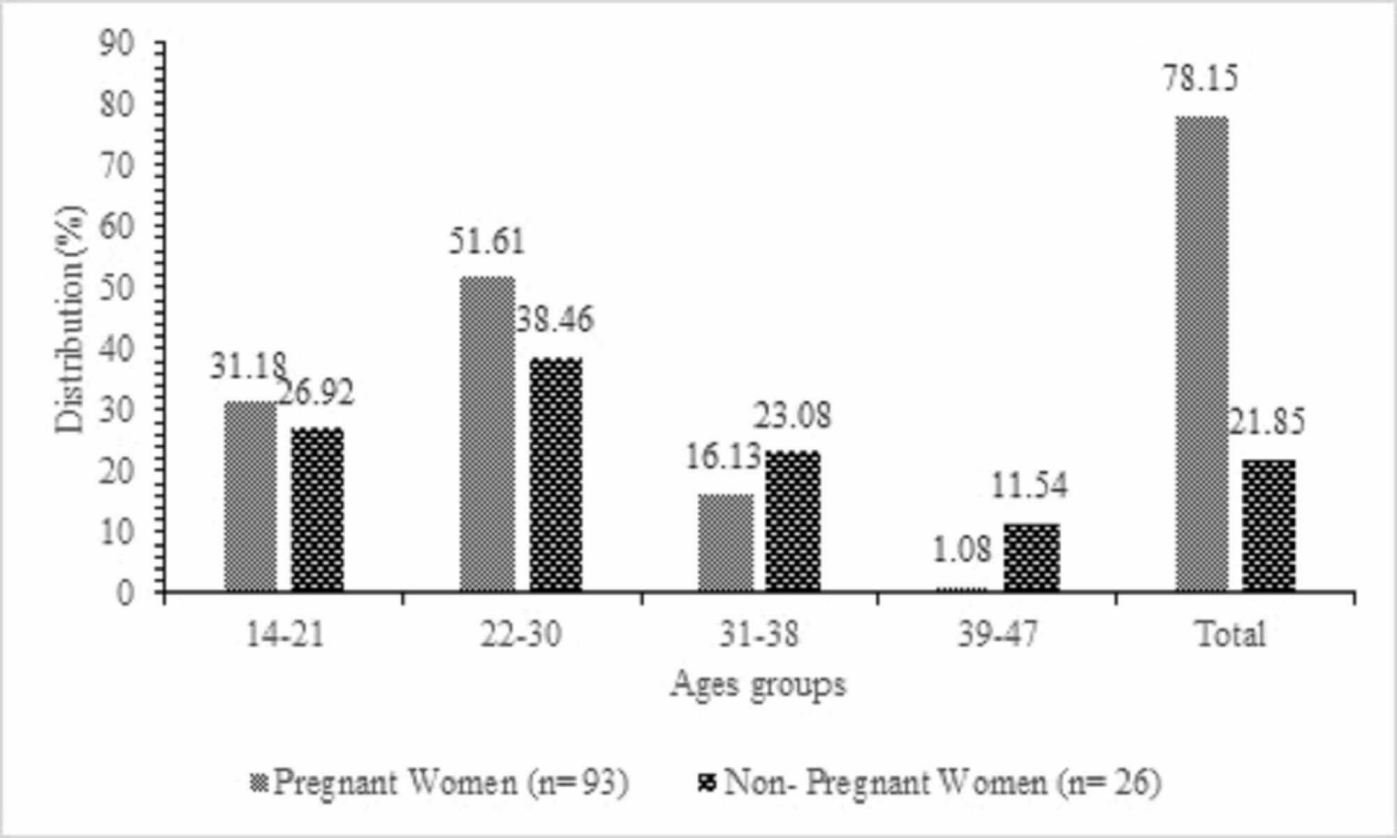
Distribution of isolated Staphylococcus aureus according to different age groups.

From 169 participants, *S. aureus* was isolated in 119 (70.41%) participants, that is, 93 (78.15%) in pregnant and 26 (21.85%) in non-pregnant women (Figure 1). On the other hand, we isolated more *S. aureus* from pregnant and non-pregnant women in the age group of 14-21 (31.18%, 26.92%) years and 22-30 years (51.61%, 38.46%), respectively.

Table 3 shows the isolation of bacteria and their association with different blood parameters. It seems that *S. aureus* were more isolated in pregnant women with a CD4 T-cell count between 410 and 625 cells/µl. More *S. aureus* were isolated from patients with serum interleukin-6 levels 25-230 (pg/ml), and CRP levels 0.2-16.8 mg/l in pregnant women with a non-significant p-value.

**Table 3:** Association between the bacterial isolates obtained and the different blood parameters measured.

Blood parameters	Range	Staphylococcus aureus (n = 119)	
		Pregnant Women (n = 93) (%)	Non-Pregnant Women (n = 26) (%)
CD4 T-cell count (Cell/µl)	193 - 409	15 (16.13)	1 (3.85)
	410 - 625	46 (49.46)	4 (15.38)
	626 - 840	23 (24.73)	10 (38.46)
	841 - 1056	9 (9.68)	11 (42.30)
		p-value < 0.001	
CD3/CD4 T-cell count (Cell/µl)	91 - 567	30 (32.26)	4 (15.38)
	568 - 1044	47 (50.54)	18 (69.23)
	1045 - 1522	16 (17.20)	4 (15.38)
		p-value = 0.357	
CD8 T-cell count (Cell/µl)	12 - 276	46 (49.46)	15 (57.70)
	277 - 541	24 (25.80)	9 (34.61)
	542 - 806	23 (6.38)	2 (7.69)
	p-value	0.508	
IL-6 count (pg/ml)	25 - 230	84 (90.32)	24 (92.30)
	231 - 435	8 (8.60)	2 (7.70)
	436 - 641	1 (1.08)	0 (0.00)
		p-value = 0.743	
hs-CRP count (mg/l)	0.2 - 16.8	91 (97.85)	25 (96.15)
	16.9 - 33.5	1 (1.08)	1 (3.85)
	33.6 - 50.2	1 (1.08)	0 (0.00)
		p-value = 0.772	

The susceptibility of the isolates obtained to eight different antibiotics was assessed in this study. Table 4 below shows the susceptibility results of the isolates of *Staphylococcus aureus* to these antibiotics. From this table it can be seen that *S. aureus* were more susceptible to imipenem (IPM) (91.40%), (100%); ciprofloxacin (CIP) (65.59%), (69.44%); amikacin (AMI) (96.77%), (100%) and resistant to chloramphenicol (CHL) (78.49%), doxycycline (DOX) (64.52%) and cefotaxime (CEFO) (51.61%) in pregnant women. In non-pregnant women *S. aureus* were more resistance to CHL (84.61%), (57.14%); DOX (69.23%), (57.14%); ERY (65.38%), (50.00%); and CEFO (69.23%), (42.86%).

**Table 4:** Antibiotic resistance profile of bacterial isolates from pregnant and non-pregnant women. IPM: Imipenem; CIP: Ciprofloxacin; CHL: Chloramphenicol; DOX: Doxycycline; AMI: Amikacin; VAN: Vancomycin; ERY: Erythromycin; CEFO: Cefotaxime; R: Resistant; I: Intermediate; S: Susceptible.

		Staphylococcus aureus (n = 119)		
Antibiotics		Pregnant Women (n = 93) (%)	Non-Pregnant Women (n = 26) (%)	p-value (between pregnant and non-pregnant)
IPM	R	4 (4.30)	1 (3.85)	0.217
	I	4 (4.30)	1 (3.85)	
	S	85 (91.40)	24 (92.30)	
CIP	R	21 (22.58)	6 (23.07)	0.560
	I	11 (11.82)	2 (7.69)	
	S	61 (65.59)	18 (69.23)	
CHL	R	73 (78.49)	22 (84.61)	0.634
	I	11 (11.82)	2 (7.69)	
	S	9 (9.68)	2 (7.69)	
DOX	R	60 (64.52)	18 (69.23)	0.186
	I	0 (0.00)	0 (0.00)	
	S	33 (35.48)	8 (30.77)	
AMI	R	2 (02.15)	1 (3.85)	0.711
	I	1 (11.82)	0 (0.00)	
	S	90 (96.77)	25 (96.15)	
VAN	R	29 (31.18)	6 (23.07)	0.695
	I	27 (29.03)	5 (19.23)	
	S	37 (39.78)	15 (57.69)	
ERY	R	46 (49.46)	17 (65.38)	0.533
	I	28 (30.10)	5 (19.23)	
	S	19 (20.43)	4 (15.38)	
CEFO	R	48 (51.61)	18 (69.23)	0.137
	I	29 (31.18)	3 (11.54)	
	S	16 (17.20)	5 (19.23)	

**Figure 2:**
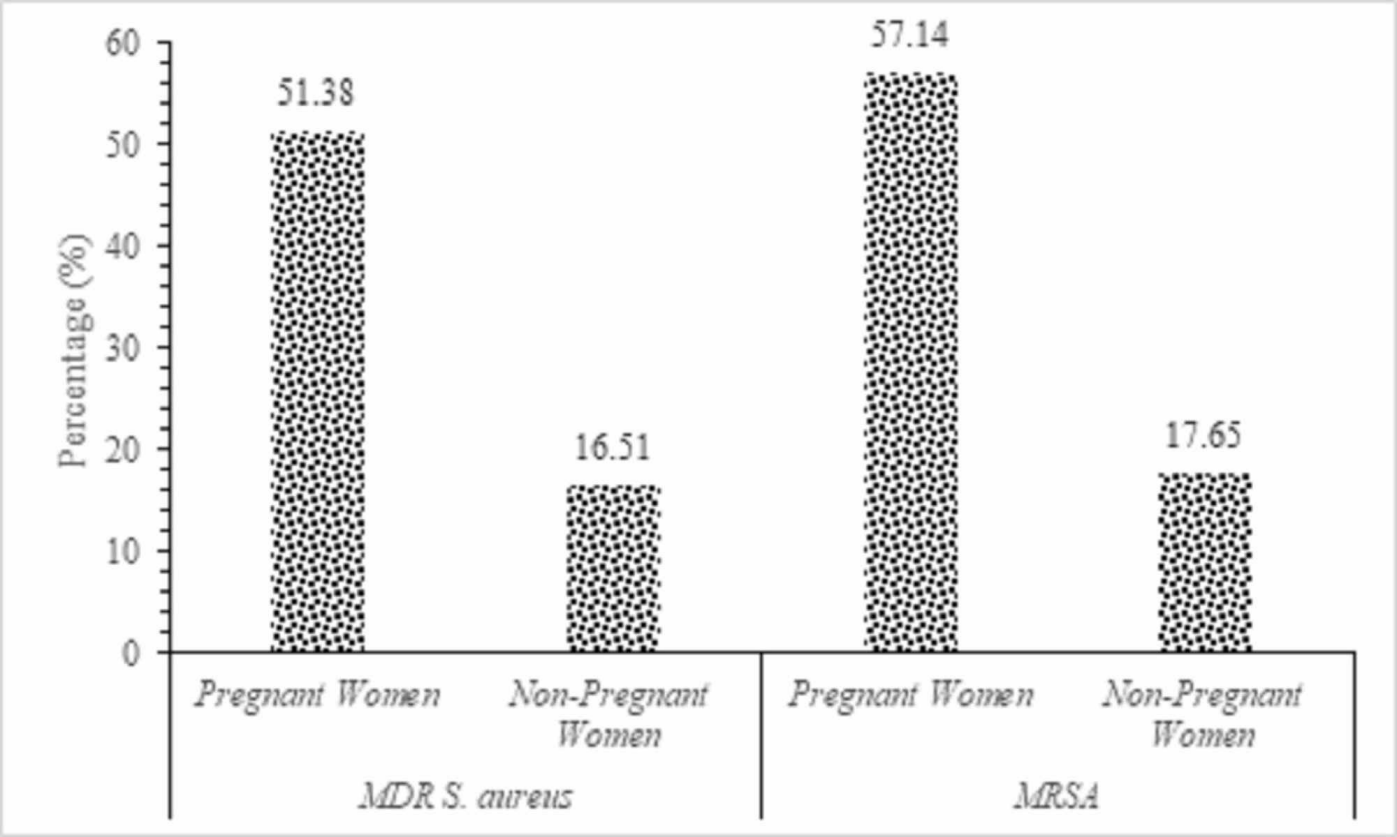
Frequency of occurrence of multidrug resistant (MDR) and Methicillin-resistant Staphylococcus aureus (MRSA) bacteria isolated from pregnant and non-pregnant women.

Figure 2 shows the frequency of occurrence of multi-drug resistance of different isolates in pregnant and non-pregnant women. All bacteria show multi-drug resistance in pregnant women compared to non-pregnant. It can be seen from the figure that isolates of *Staphylococcus aureus* showed a significant increased multi-drug resistant (MDR) and Methicillin-resistant *Staphylococcus aureus* (MRSA) in pregnant compare to non-pregnant women (p < 0.05).

## Discussion

Methicillin-resistant *Staphylococcus aureus* (MRSA) causes a range of illnesses, from skin and wound infections to pneumonia and bloodstream infections that can cause sepsis and death [12]. This infection is increasing among pregnant and postpartum women. *Staphylococcus aureus*, including MRSA, are one of the most common nosocomial and community-acquired pathogens, a cause of healthcare-associated infections [13]. It has now emerged as an ever-increasing problem due to its increasing resistance to several antibiotics. This study determined the susceptibility pattern of *S. aureus* strains isolated from stool of pregnant and non-pregnant women in different hospitals of Bafang to provide physicians with up-to-date information about the local data of antimicrobial resistance of this pathogen.

Out of the 169 patients studied, 76.30% patients were pregnant women and 23.70% were non-pregnant women. The significant difference in the distribution of the different age groups was observed. The mean age of the participants was significantly higher in non-pregnant women (29.38 ± 7.685 years) compared to pregnant women (25.55 ± 5.521 years). Results of the blood parameters showed that CD4 T-cell was significantly lower in pregnant than non-pregnant women, contrary for CD8 T-cell. Total white blood cells, granulocytes, were higher but non-significant in pregnant women compared to those who were non-pregnant. CD4 T-cells and CD8 T-cells counts are widely used prognostic markers to assess the degree of immune system. Pregnancy is considered as a physiologically immunocompromised state, hence alterations in T-lymphocyte subsets may occur during pregnancy. There is need to establish baseline values of these counts, especially in healthy pregnant women [14]. This study shows that CD4 T-cells were significantly lower in pregnant than non-pregnant women, respectively. This result is similar than the one of Ekwempu et al. in 2012 who showed a progressive decline in the CD4 count of the HIV-negative women, from 610 cells per µl to 534 cells per µL (P < .05), which was not significant. This finding may be associated with other factors, such as stress or decreased immunity [15].

From 169 participants, *S. aureus *was isolated in 70.41% participants, that is, 78.15% in pregnant and 21.85% in non-pregnant women. More *S. aureus* were isolated from pregnant and non-pregnant women in the age group of 14-21 (31.18%, 26.92%) years and 22-30 years (51.61%, 38.46%), respectively. This is in agreement with previous studies which had a similar finding [16,17]. However, the occurrence of 78.15% in pregnant women and 21.85% in non-pregnant women seen in our study was much higher than the 22.8% obtained by Akortha and Ibadin in 2008 [18].

In this study, it was equally observed that *S. aureus* were more isolated in patients with a CD4 T-cell count between 410 and 625 cells/μl, while CD8 T-cell count between 12 and 276 cells/μl and with a non-significant p-value. The T-cell suppression in chronic *S. aureus* infection was documented and attributed to myeloid-derived suppressor cells (MDSCs) with a minor contribution from Tregs [19,20]. Towers et al. also reported a mean absolute CD8+ cell count that was not significantly different and therefore appears to be unaffected during pregnancy [21]. Therefore, regional diversity in the T-lymphocyte subset count is evident. In addition to the regional changes, variations could also be due to use of different equipment and techniques in different studies and could have given rise to procedural and instrumental errors. In contrast, more bacteria were isolated from patients with serum interleukin-6 levels 25-230 pg/ml, and CRP levels 0.2-16.8 mg/l in pregnant women with a non-significant p-value. IL-6 is a multifunctional cytokine that plays a key role in the inflammatory response and in the direction of T-cell differentiation in adaptive immunity [22]. IL-6 is widely expressed in the female reproductive tract and gestational tissues thus, exerting regulatory functions in embryonic implantation and placental development, as well as the immune adaptations necessary to tolerate pregnancy [23].

Results of antimicrobial susceptibility testing revealed that *Staphylococcus aureus* were more susceptible to IPM, CIP and AMI in pregnant women. In non-pregnant women, isolates of *Staphylococcus aureus* were more resistance to CHL, DOX, ERY and CEFO with no statistically significant difference in the distribution in the pregnant as well as in the non-pregnant women. This study also showed a high rate of MDR and MRSA which seems to be similar to findings of 65.7% by Asiimwe et al. [24]. It has been noticed that the proportion of MRSA has increased worldwide since the past two decades and the rate varies markedly across different countries and among hospitals of the same country [25]. Improper infection prevention practices in the hospital set up, indiscriminate use of antibiotics, intravascular catheterizations, hospitalization in intensive care unit contribute in the emergence of MRSA [26]. These factors as well as the difference in the study population may explain these variations.

The data of this study might not be representative of the entire pregnant women; however, they could be of interest to physicians and other health professionals. Because of a limited number of participants involved in the present work, further studies will be performed to increase the reliability of these findings. The cross-sectional design of this study limits the ability to address causal relationships between variables.

## Conclusions

The present study revealed that the distribution of *Staphylococcus aureus* infections, as well as resistant phenotypes, was higher in pregnant women. The immunological anomalies observed in pregnant women probably caused the pregnancy status. Hence, the resistance profiles of *Staphylococcus aureus* should be considered in pregnant women to allow better follow-up during pregnancy. However, this work also indicated the need for adequate immunological follow-up for the best monitoring of pregnant women.
